# Small archaea may form intimate partnerships to maximize their metabolic potential

**DOI:** 10.1128/mbio.00347-24

**Published:** 2024-08-29

**Authors:** Brett J. Baker, Natalie Sarno

**Affiliations:** 1 Department of Integrative Biology, University of Texas at Austin, Austin, Texas, USA; 2 Department of Marine Science, Marine Science Institute, University of Texas at Austin, Port Aransas, Texas, USA; Oregon State University, Corvallis, Oregon, USA

**Keywords:** DPANN archaea, oxygen deficient zones, marine nutrients, Archaea, metagenomics, nitrogen cycling

## Abstract

DPANN archaea have characteristically small cells and unique genomes that were long overlooked in diversity surveys. Their reduced genomes often lack essential metabolic pathways, requiring symbiotic relationships with other archaeal and bacterial hosts for survival. Yet a long-standing question remains, what is the advantage of maintaining ultrasmall cells. A recent study by Zhang et al. examined genomes of DPANN archaea from marine oxygen deficient zones (ODZs) (I. H. Zhang, B. Borer, R. Zhao, S. Wilbert, et al., mBio 15:e02918-23, 2024, https://doi.org/10.1128/mbio.02918-23). Surprisingly, these genomes contain a broad array of metabolic pathways including genes predicted to be involved in nitrous oxide (N_2_O) reduction. However, N_2_O levels are likely too low in ODZs to make this metabolically feasible. Modeling co-localization of DPANN archaea (N_2_O consumers) with other larger cells (N_2_O producers) demonstrates that N_2_O uptake rates can be optimized by maximizing the producer-to-consumer size ratio and proximity of consumer cells to producers. This may explain why such a diversity of archaea maintain extremely small cell sizes.

## COMMENTARY

The vastness of microbial biodiversity of our planet has come into focus through exploration of nature via DNA sequencing. Metagenomics, the ability to obtain genomes directly from natural communities, has been fueled by recent technological advances in sequencing and computational approaches. This exploration has revolutionized our understanding of microbial diversity and evolution (1, 2). Huge swathes of novel branches have been added to the tree of life, notably, the Asgard archaea (2) and the DPANN (3). The first description of a lineage that is now included in the DPANN was *Nanoarchaea equitans*. These Nanoarchaeota have tiny cells (400 nm in diameter) that hitchhike by attaching to *Ignicoccus*—a cultured hyperthermophile (4). Shockingly, the genome of *N. equitans* is only ~500 Mb in size and they were found to be a new phyla of archaea.

The first whole community metagenomic studies from an acid mine drainage site (at Iron Mountain in Northern California) yielded a DNA fragment containing a unique 16S rRNA gene (5, 6). Commonly used PCR primers did not hit this unique 16S gene so it was long overlooked in traditional 16S diversity surveys. Filtering the acid mine communities through a 450 nm filter yielded enrichments of cells containing this unique 16S. At the time, these small cells with unique 16S genes were referred to as acidophilic enrichment mine archaea nanoorganisms (ARMAN). Filtrate enrichments led to the first genomes for these archaea, now classified as Micraarchaeota and Parvarchaeota, which revealed a very small genome size (~1 Mb). Fluorescent *in situ* hybridization (FISH) and cryo-electron tomography studies revealed that the cell size was also small, testing the lower predicted limits for life (0.009 μm^3^), and further showed that these cells only contain ~90 ribosomes each (7, 8). Interestingly, even though these cells are seen to be free living, many of them were shown to have cell wall connections with other archaea in the community from the Euryarchaeota.

The diversity of DPANN has expanded greatly in the past 15 years to include over 12 phyla (9). However, their metabolic capabilities are generally limited compared to other archaea. DPANN often lacks essential biosynthetic pathways for lipids, amino acids, and nucleotides (10). Although they are almost exclusively associated with low-oxygen or anoxic environments and have been found worldwide in acidic and hypersaline ponds as well as in marine, freshwater, terrestrial, and animal microbiomes (10), their ecological roles are not well understood. DPANN are also lacking biogeochemically relevant pathways for nutrient cycling like nitrogen and sulfur metabolism.

A new study by Zhang et al. (11) examined the genomic diversity of DPANN archaea in pelagic oxygen deficient zones (ODZs) from the Arabian Sea and the eastern tropical North and South Pacific regions (ETNP and ETSP). From a set of 962 metagenome assembled genomes (MAGs), they identified 33 belonging to the DPANN phyla Nanoarchaeota, Pacearchaeota, Woesearchaeota, Undinarchaeota, Iainarchaeota, and SpSt-1190. None of these DPANN MAGs were obtained from oxygenated samples. Genomic coverage demonstrates that these DPANN were most dominant at 200 meters depth in the ETNP and Arabian Sea where they are 1% and 0.8% of the total community and 50% and 27% of the archaea, respectively. The relatively high abundance of these DPANN suggests that they are likely important in ODZ communities.

DPANN from ODZs appear to have a versatile metabolic repertoire. Like many other DPANN genomes, those recovered from ODZs have varying completeness of central carbon and biosynthetic pathways. Interestingly, the ODZ Woesearchaeota appear to encode genes for cyclopropane fatty acid phospholipid synthesis. Typically, these phospholipids are stabilizers for bacterial membranes and have never been identified in archaea before. Many of the Woesearcheaota and Undinarchaeota are capable of fermentation, and SpSt-1190 have near complete methanogenesis pathways; however, they are missing the essential methyl-coenzyme M (*mcr*) genes. The *mcr* genes are essential as these genes encode methyl-coenzyme M reductase, the only known enzyme that catalyzes the final step of methanogenesis, methane formation. Thus, it is possible that these DPANN may utilize the annotated methanogenesis genes in other metabolic pathways that do not result in the production of methane. Perhaps most notable among these genomes is the presence of nitrous oxide reductase genes (*nosZ*), which are predicted to be involved in N_2_O reduction to N_2_. A phylogeny of these DPANN NosZ-like proteins revealed that they are distinct from homologous cytochrome c oxidase subunit II and clade II Sec-type NosZ. Zhang et al. (11) attempted to heterologously express three of the DPANN *nosZ*-like genes in *Pseudomonas aeruginosa*. They were unable to confirm any significant N_2_O reduction in the mutants compared to the *P. aeruginosa* native *nosZ* complementation.

As N_2_O is present at low nanomolar concentrations in ODZ waters, there may not be enough for any microbes to make a living from its consumption. Zhang et al. (11) explore an alternative scenario where high levels of N_2_O may localize near cells that produce N_2_O such that the characteristically small DPANN may be able to get close enough to these N_2_O producers to survive. In this study, they predict N_2_O reduction feasibility by simulating cell size ratios and distance between cells. When the N_2_O consumer is small relative to the N_2_O producer, and when the distance between the two is small, the reduction of N_2_O is metabolically favorable. Thus, as the consumer-to-producer cell size ratio decreases the uptake rate, normalized to cell volume, increases enabling the N_2_O consumer to obtain more substrate. Absolute concentrations of N_2_O with respect to the consumer also increase furthering substrate acquisition even more. The substrate uptake rate differences are dramatic. The example provided by Zhang et al. (11) demonstrates that, as the consumer-to-producer radii ratio increases from 0.1 to 1, there is a 100-fold decrease in N_2_O uptake for attached consumer cells. While it has already been assumed that DPANN archaea and candidate phyla radiation (CPR) bacteria (small symbiotic bacteria) maintain small cells to have lower energy requirements, this modeling provides another view on why being small may be advantageous. This study provides a plausible explanation as to why there is such a broad diversity of microbes (CPR and DPANN) that are significantly smaller than their hosts.
